# Province Disparity on Female Breast Cancer in Iran

**Published:** 2017-12

**Authors:** Erfan AYUBI, Ali HOSSEINI, Kamyar MANSORI, Salman KHAZAEI

**Affiliations:** 1. Dept. of Epidemiology, School of Public Health, Shahid Beheshti University of Medical Sciences, Tehran, Iran; 2. Dept. of Epidemiology and Biostatistics, School of Public Health, Tehran University of Medical Sciences, Tehran, Iran; 3. Dept. of Geography and Urban Planning, University of Tehran, Tehran, Iran; 4. Social Determinants of Health Research Center, Kurdistan University of Medical Sciences, Sanandaj, Iran; 5. Dept. of Epidemiology, School of Public Health, Hamadan University of Medical Sciences, Hamadan, Iran

## Dear Editor-in-Chief

The cancer statistics in Iran showed that breast cancer has increased in last two decades; therefore, it becomes most common cancer that affects women. Breast cancer has first rank and fifth rank among cancer incidence and cancer mortality in Iranian women respectively (1, 2). In other hand predicted models have demonstrated breast cancer incidence in women would be three times (3). Therefore, breast cancer in women can be considered as one of the health priorities in Iran.

An inequality cancer burden is borne by low socioeconomic status (SES) provinces and these provinces have higher incidence rates (4). For cancer, control and prevention activities, health policies must target on high-risk regions. Then information about spatial patterns can lead to better health policies for reducing breast cancer in Iranian women.

We motivated to find high rate clusters of breast cancer in Iranian women using by cancer provinces data from 2008 national registry of cancer (NCR) in Iran. Women population of each province was obtained by the 2006 national census data from Statistical Centre of Iran.

**Fig. 1:**
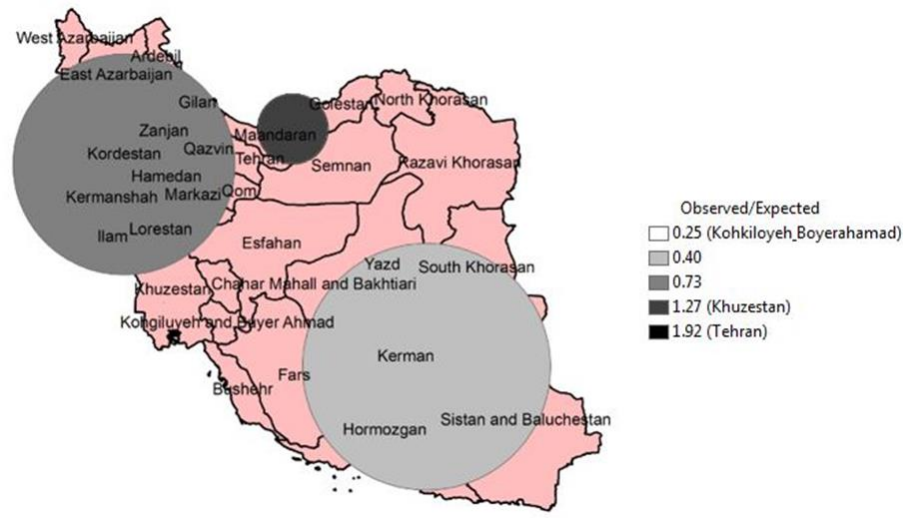
High rate clusters of female breast cancer in Iran, 2008

We addressed this objective by Kulldorff spatial scan statistic in SaTScan software. As shown in Fig. 1, the provinces that located in Southeastern and Northwestern Iran had lower rates of breast cancer while provinces located in central and northern of Iran had higher rate breast cancer.

Tehran and Kohgiluyeh and Boyer-Ahmad provinces have highest and lowest ratio of observed/expected (obs/exp) breast cancer obs/exp=1.92 and obs/exp=0.25 respectively. Breast cancer in women differently distributed across provinces and province based characteristics such as SES can impede this geography disparity. The results are relevant for answering which provinces can be targeted for screening and health care allocation and policy decisions.
